# Multi-view carotid ultrasound is stronger associated with cardiovascular risk factors than presence of plaque or single carotid intima media thickness measurements in subclinical atherosclerosis

**DOI:** 10.1007/s10554-023-02868-0

**Published:** 2023-05-30

**Authors:** Anna Bengtsson, Emma Nyman, Christer Grönlund, Per Wester, Ulf Näslund, Eva Fhärm, Margareta Norberg

**Affiliations:** 1 Department of Epidemiology and Global Health, Umeå University, Umeå, S-901 87 Sweden; 2 Unit of Family Medicine, Department of Public Health and Clinical Medicine, Umeå University, Umeå, Sweden; 3 Unit of Medicine, Department of Public Health and Clinical Medicine, Umeå University, Umeå, Sweden; 4 Department of Radiation Sciences, Biomedical engineering, Umeå University, Umeå, Sweden

**Keywords:** Atherosclerosis, Cardiovascular disease, Carotid intima media thickness, Carotid plaque, Carotid ultrasound

## Abstract

We aimed to explore the prevalence of atherosclerosis by using multi-view ultrasound examination of the carotid arteries and its association with clinical risk factors in a middle-aged population at low to intermediate risk of cardiovascular disease (CVD). Carotid vascular ultrasound was performed in 3532 participants in the VIPVIZA trial. Mean and maximal carotid intima media thickness (cIMT) at prespecified angles and plaque presence were examined on the left and right side. Associations between CVD risk factors and ultrasound variables were quantified by partial least squares (PLS) regression. A combined ultrasound variable was computed using weights of the first PLS component. Associations between CVD risk factors and the combined multi-view ultrasound variable, single cIMT and plaque measurements, respectively, were determined using linear regression modelling. The participants’ mean age was 55.7 years and 52.9% were women. Plaque prevalence was 51.1% in men and 39.0% in women. cIMT was higher in men than in women and in the left compared with the right carotid artery. The strongest association of CVD risk factors was observed with the combined multi-view ultrasound variable (R2 = 24%), compared with single cIMT variables (R2 = 14–18%) and plaque presence (R2 = 15%). The pattern was similar in both sexes. The association with CVD risk factors and the combined ultrasound variable was stronger in 40-year olds (R2 = 22%) compared with 50- or 60-year olds (R = 12%). CVD risk factors are stronger associated with a combined ultrasound variable than plaque presence or single cIMT measures suggesting that carotid multi-view ultrasonography better captures the focality of early atherosclerosis.

Clinical Trial Registration: ClinicalTrials.gov, number NCT01849575. May 8, 2013.

**Supplementary Information:**

The online version contains supplementary material available at 10.1007/s10554-023-02868-0.

## Introduction

Atherosclerosis is by far the most important cause of cardiovascular disease (CVD) which accounts for most deaths and disability worldwide [1]. Reduction of risk factors can curb the progression of atherosclerosis and decrease the risk of CVD events [2, 3]. Detection of early subclinical atherosclerosis to identify individuals at increased risk of CVD can be the basis for early risk-reducing actions to prevent or delay future CVD development.

To detect and measure early subclinical atherosclerosis carotid B-mode ultrasound examination can be useful. Increased carotid intima media thickness (cIMT) and progression of atherosclerosis are associated with the same major CVD risk factors [4, 5]. Carotid IMT has been suggested as a marker of vascular ageing [6, 7] while carotid plaque, as an indicator of subclinical atherosclerosis, has demonstrated stronger predictive ability of future CVD [8, 9]. However, many studies that evaluate the potential to predict CVD are performed in populations at high risk of CVD [10].

Comparing different studies regarding plaque prevalence and cIMT is challenging due to different plaque definitions and inconsistent protocols for measuring cIMT [11, 12]. To use semi-automatic equipment with standardized protocols may facilitate the reproducibility of examinations [11]. In subclinical atherosclerosis, there is a subtle and heterogeneous thickening of the carotid wall. To detect this focality and cover the degree of the atherosclerotic burden, a multi-view screening, including both left and right side and also differences within each side, is recommended [13–16]. Moreover, populations with different degree of atherosclerosis may not be comparable regarding associations between risk factors, ultrasound variables, and hard end-points. While early atherosclerosis is likely to have a long latency before a clinical event, severe atherosclerosis demonstrates the opposite. Population statistics for cIMT, carotid plaque and CVD risk factors often miss detailed study population characteristics, therefore specific determinants and interactions in the development of atherosclerosis are difficult to evaluate when comparing populations. Most studies are performed on advanced atherosclerosis, therefore there is a need for studies on early atherosclerosis.

Previous research has shown associations between atherosclerosis and various risk factors, but with a relatively low degree of explanation [17–19]. Here we hypothesize that sampling the atherosclerosis in both carotid arteries at several projections with cIMT and plaque measurements, may better capture the focally heterogenic atherosclerotic disease in its early stage, as compared to single cIMT and plaque measurements. A combination of these ultrasound measurements may thereby also associate more strongly with CVD risk factors.

The aim of this study was primarily to describe the prevalence of atherosclerosis as assessed by carotid ultrasound in a middle-aged population at low/intermediate risk of CVD and secondly to investigate the association between clinical risk factors and ultrasound variables.

## Materials and methods

### Setting, design and study population

Individuals in this study were participants in the VIPVIZA trial. VIPVIZA is the acronym for **VI**suali**Z**ation of asymptomatic **A**therosclerotic disease for optimum cardiovascular prevention ─ a randomized controlled trial nested in the **V**ästerbotten **I**ntervention **P**rogram (VIP). In VIP, inhabitants in Västerbotten county are invited to their primary care center the years they turn 40, 50 and 60 years old for CVD risk factor screening and individual counseling aiming at prevention of CVD and diabetes. Participants were informed and invited to VIPVIZA at the occasion of participation in VIP. The inclusion and the baseline ultrasound examinations were performed between April 29 2013 and June 7 2016.

Inclusion criteria for VIPVIZA were (i) Age 40 and family history of CVD before age 60 among first-degree relatives. (ii) Age 50 and at least one of the following risk factors: first-degree relative with CVD before age of 60, smoking, diabetes, hypertension, s-LDL cholesterol ≥ 4,5 mmol/l, abdominal obesity. (iii) Age 60. In case of significant carotid stenosis (> 50% by NASCET based on ultrasound Doppler measurement of peak systolic velocity ≥ 1.5 m/s), the participant was not included in the study and was referred to special care (n = 22).

This paper reports cross-sectional analyses of baseline data collected at the VIP visit and the baseline ultrasound results [20].

### Clinical risk factors and socioeconomic status

Measurements of clinical risk factors were standardized and performed according to the VIP protocol [21]. Height was measured with light clothing and without shoes. Weight was measured with a calibrated scale. Body mass index (BMI) was calculated as weight/(height)^2^. Waist circumference was measured in cm with the subject in a standing position after a slight exhalation. Blood pressure was measured twice with subject sitting and after 5 min rest with a precision of 2 mm; the mean of both the systolic and the diastolic values were recorded. Blood samples were drawn after overnight fasting. Oral glucose tolerance testing was performed according to WHO-standards [22]. Total-cholesterol (TC), high-density lipoprotein-cholesterol (HDL), and triglycerides (TG) were analyzed with routine clinical methods at the nearest hospital. LDL-cholesterol (LDL) was calculated with Fridewald’s formula [23]. Diabetes mellitus (DM) was defined as fasting glucose ≥ 7.0 mmol/l, or 2-hour-glucose ≥ 12.2 mmol/l (capillary plasma) or self-reported previous diagnosis of diabetes.

Questionnaires were used to record self-reported lifestyle habits; Smoking (never, former, current), Snuffing (Yes/No), Educational level (Basic level: up to 9 schooling years, Mid-level: 10 − 12 schooling years, and University/Academic level: ≥ 13 schooling years), Fruit and vegetable consumption (fruits or berries more than 2–3 times/day and vegetables at least 2–3 times/days added up to the general recommendation of ≥ 500 g /day: Yes/No), Physical activity (fulfilling general recommendations for physical activity: ≥150 min/week moderate physical intensity and/or ≥ 75 min/week high physical intensity: Yes/No). In addition, self-reported Use of anti-hypertensive medication (Yes/No), Use of lipid lowering medication (Yes/No), Previous hospitalization for a verified heart attack/myocardial infarction (Yes/No), Geographical region for residency categorized into Urban including Umeå city region > 150,000 inhabitants, and Rural with towns (< 100,000 inhabitants), villages and countryside.

### Ultrasound examination and variables

Bilateral carotid artery ultrasound scan was performed with the portable ultrasound system Panasonic CardioHealth® Station (Panasonic Healthcare Corporation of North America, Newark, NK, USA) with a linear 7 MHz transducer. All ultrasound scans were performed by specially trained sonographers according to the same standardized protocol throughout the study [20], where the common carotid artery (CCA), bifurcation, and internal and external carotid arteries were examined. Time reserved for each ultrasound scan was 15 min. Carotid plaques were identified according to Mannheim plaque consensus [24] in the near and far walls and decision of plaque occurrence was taken during the time of examination.

**Fig. 1:**
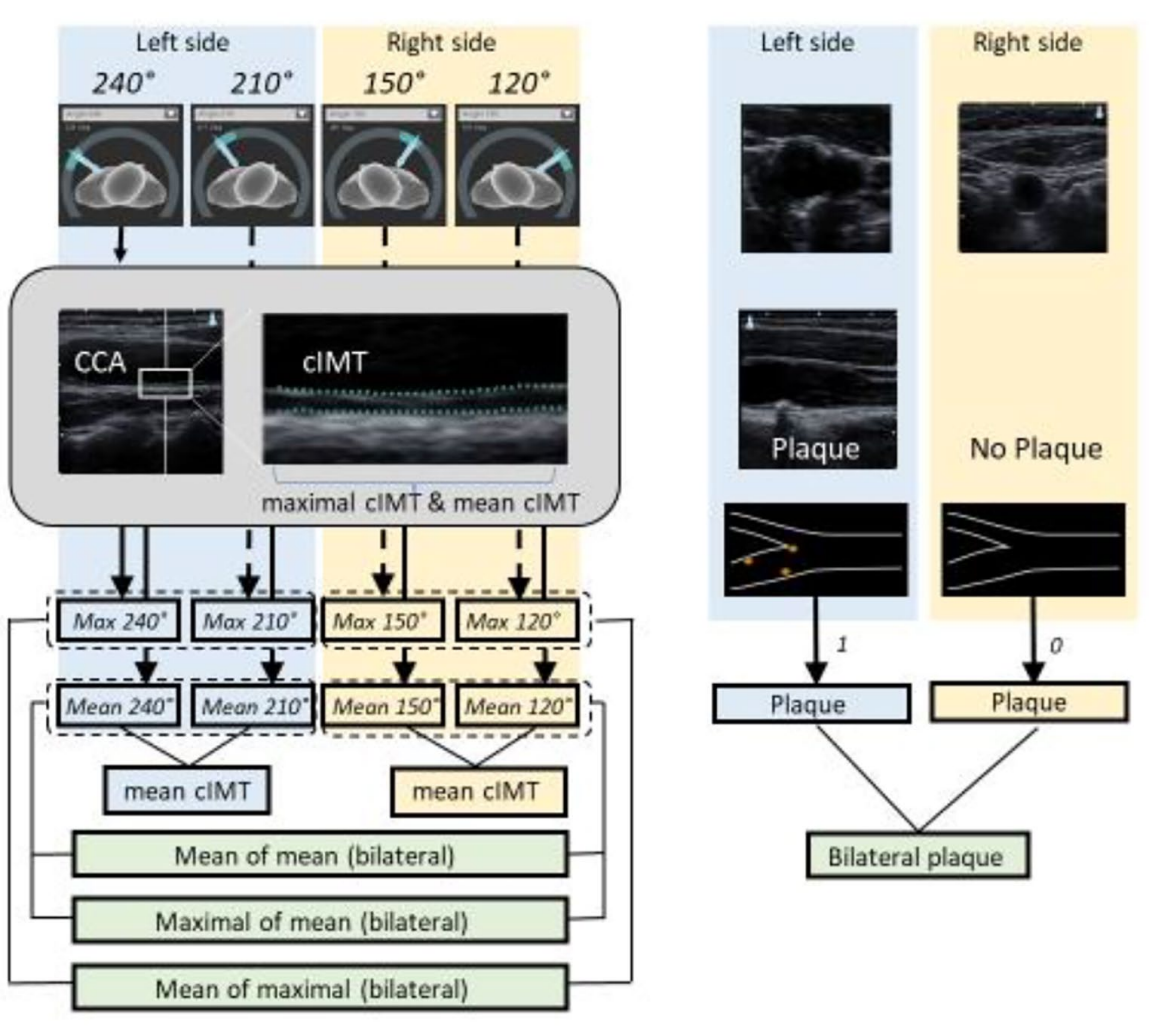
Illustration of the ultrasound carotid intima media thickness (cIMT) measurements. Both carotid arteries of each participant were scanned at two standardized angles (120 and 150 vs. 210 and 240). In each projection (angle), the cIMT was automatically measured by the mean of mean cIMT and max of mean cIMT. Additionally, the maximal mean value of all four angles were selected and titled ‘maximal mean value’

Real-time automatic edge-detection measurement of cIMT was performed in the distal 1-cm of the far wall of CCA at two predefined angles for insonation at each side (240°, 210° and 150°, 120° for left and right carotid artery, respectively) based on Meijer arc. For each angle, mean and max cIMT were measured and reported in a 10 mm plaque free segment. (Fig. 1)

Based on the cIMT measurements from each angle from both sides, bilateral mean of mean and mean of maximal value was calculated. Additionally, the bilateral maximal mean value of all four angles was selected and titled maximal mean value. Further, mean and maximal values measured at the left and right sides separately, were used. All 16 ultrasound variables are shown in Table 2.

All measurements were performed at end diastole by automatic detection of temporal lumen diameter changes. The reproducibility of plaque detection as plaque present or not present and cIMT measurement has been published previously and the Kappa value of plaque detection was 0.70 (95% CI 0.60–0.80) for the inter-sonographer variability [25]. The mean and standard deviation of the inter-sonographer variability of cIMT measurement was 0.015 (± 0.079)mm, with a intraclass correlation of coefficient of 0.95 [26].

### Statistical analyses

Clinical risk factor measurements as well as ultrasound variables were reported as means and SD for continuous variables and distributions for categorical variables.

**Fig. 2:**
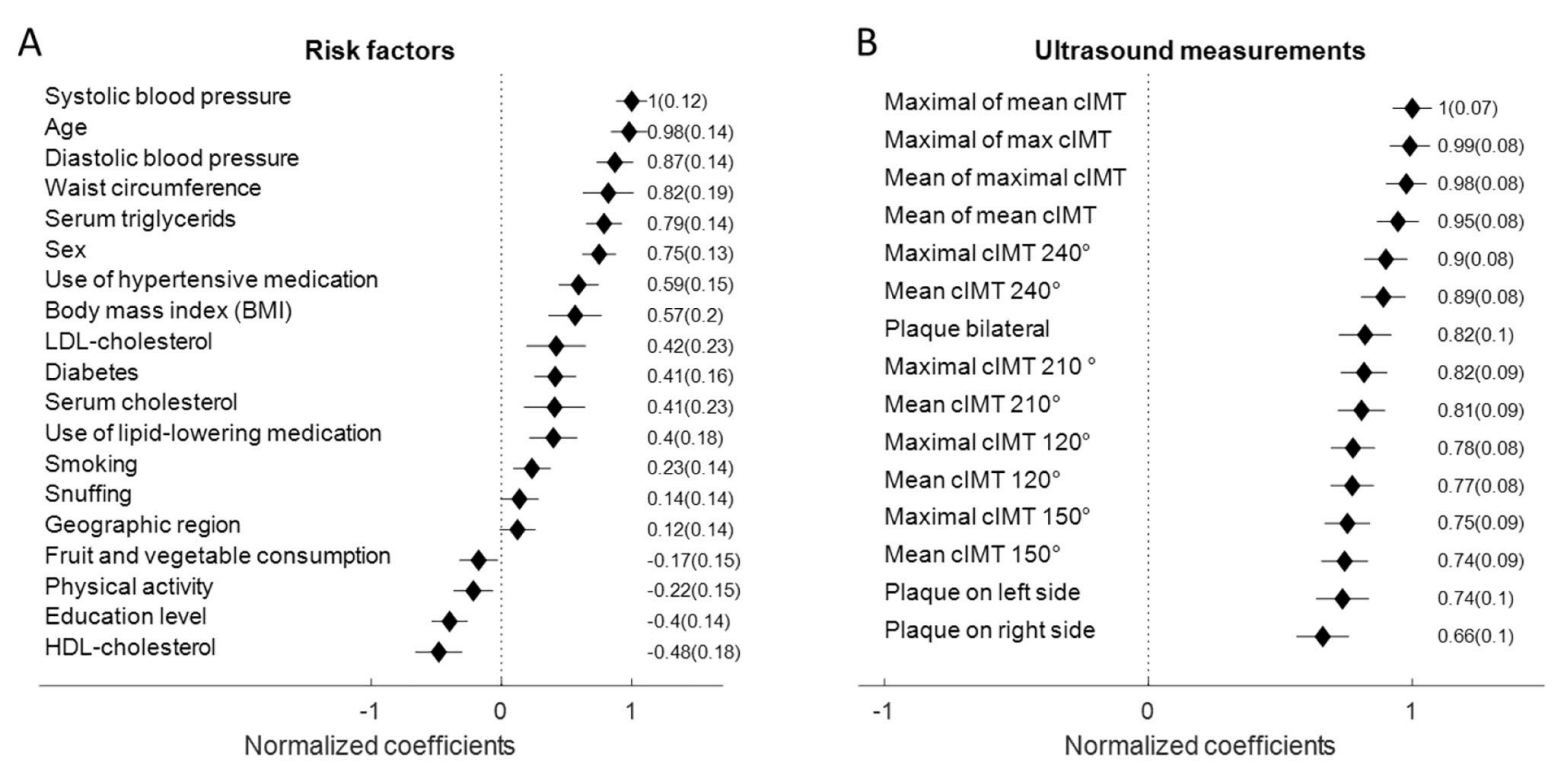
Associations between risk factors and ultrasound variables quantified by the weights of the PLS regression analysis between clinical risk factors (A) and ultrasound variables (B). The PLS regression analysis provide the weights (A) how to combine the ultrasound variables such that a weighted combination (B) of the risk factors can explain their variance maximally. The Mean and Standard deviation of the weights of the regression model are presented

The overall associations between the two data sets (risk factors and ultrasound variables) were explored using partial least squares (PLS) regression analysis [27]. This analysis uses the multivariate variance of each data set to find projections where an optimal linear regression fit can be obtained (Supplementary Fig. 1). Ultrasound variables were input as response variable (Y) and risk factors as explanatory variable (X). In practice, the PLS regression analysis provide the weights how to combine the ultrasound variables such that the risk factors explain their variance maximally. In this way we both get regression weights on how the X variables should be combined, and simultaneously, how the Y variables should be combined. Associations between risk factors and ultrasound variables were quantified by the weights of the PLS regression analysis between ultrasound variables and clinical risk factors. Mean and standard deviation of the weights of the regression model are presented. (Fig. 2). The age variable was treated as an ordinal categorical variable. The results of the first PLS component of the ultrasound PLS component, defined as Combined ultrasound measurement, includes all weighted cIMT variables and plaque (combination of cIMTs and plaque Y/N) and was used in this work. A bootstrapping procedure was used to compute the weights, using 500 randomly selected samples and 500 iterations. This provided mean and standard deviation to the weights of all variables in X and Y.

Next, significant associations between risk factors and ultrasound variables were determined using step-wise linear regression modelling. The following models were tested:

Model 1: Combined ultrasound measurement vs. risk factors.

Model 2: Bilateral maximal mean cIMT vs. risk factors. This variable was included since it was used in the VIPVIZA study to evaluate vascular age. Due to the heterogeneity of cIMT during early atherosclerosis, the most unfavorable mean value was translated to vascular age in the pictorial presentation received by the intervention group [20].

Model 3: Presence of plaque vs. risk factors.

Model 4: Right mean cIMT vs. risk factors. This variable was chosen because other studies have used it, for example the Tromsö study [28] and the MESA study [29].

Model 5: Left mean cIMT vs. risk factors. This variable was used due to slightly higher cIMT values on the left side.

Model 6 and 7: Investigation of sex differences. Combined ultrasound measurement vs. risk factors, men and women separately.

Model 8–10: Investigation of age differences. Combined ultrasound measurement vs. risk factors by ages 40, 50 and 60 years.

The PLS and stepwise linear regression modelling was done in MATLAB (2018b, Mathworks, Nattick, MA, USA) and the descriptive statistics were carried out using SPSS Statistics 24 software (IBM Corporation, Armonk, NY, USA).

## Results

### Study population

Among 4177 VIP participants who fulfilled the clinical risk factor-based inclusion criteria and were invited to VIPVIZA, 345 declined to participate. Among those 3832 who consented to participation, 121 withdrew their consent between VIP participation and the baseline ultrasound examination, 154 dropped out, twenty-two had significant stenosis and were excluded, and three died before the ultrasound examination. Thus, 3532 participants were included in the trial. The drop-out analysis showed some differences between participants and drop-outs. Among drop-outs total cholesterol, LDL-cholesterol and diastolic blood pressure were lower and triglyceride levels and smoking prevalence were higher. Overall, the differences between participants and drop-outs were small and point in different directions with regard to CVD risk. (Supplementary Table 1)

Baseline characteristics (Tables 1 and 2): The population comprised 52.9% women and mean age was 55.5 and 55.8 years among men and women respectively; 12.4% of men and 12.9% of women were smokers. The systolic blood pressure was 132 and 127 mmHg in men and women. Among men, 8.4% had diabetes vs. 5.8% among women.

**Table 1:** Baseline characteristics among men and women in the VIPVIZA trial

Characteristics	Men (n = 1662)	Women (n = 1870)	Missing n (%)	Total (n = 3532)
Age mean (SD)	55.5(6.41)	55.8(6.27)	0	55.7(6.39)
Smoking ^1^			8(0.2)	
Never smoked n (%)	886(53.4)	908(48.7)		1794(50.9)
Ex-smoker n (%)	567(34.2)	717(38.4)		1284(36.4)
Smoker n (%)	205(12.4)	241(12.9)		446(12.7)
Education level^1^			35(1.0)	
Basic n (%)	166(10.1)	157(8.5)		323(9.2)
Mid-level n (%)	1023(62.0)	934(50.6)		1957(56.0)
University n (%)	462(28.0)	755(40.9)		1217(34.8)
Use of hypertensive medication^1^ n (%)	522(32.5)	534(29.6)	123(3.5)	1056(31.0)
Use of lipid lowering medication^1^ n (%)	235(14.7)	155(8.6)	123(3.5)	390(11.4)
Previous myocardial inf ^1^ n (%)	60(3.6)	17(0.9)	30(0.1)	77(2.2)
Age group			0	
40 years n (%)	136(8.2)	140(7.5)		276(7.8)
50 years n (%)	472(28.4)	506(27.1)		978(27.7)
60 years n (%)	1054(63.4)	1224(65.5)		2278(64.5)
Fruit and vegetable consumption n (%)	354(21.3)	786(42.2)	10(0.3)	1140(32.4)
Physically active n (%)	887(53.7)	1103(59.6)	29(0.8)	1990(56.8)
Snuffing daily/occasionally n (%)	450(27.5)	184(10.0)	61(1.7)	634(18.3)
Region			10(0.3)	
Umeå- City n (%)	682(41.1)	783(42.0)		1465(41.6)
Rural n (%)	977(58.9)	1080(58.0)		2057(58.4)
Serum triglycerides mmol/l mean (SD)	1.67(1.13)	1.33(0.72)	1(0.0)	1,49(0.95)
LDL-cholesterol mmol/l mean (SD)	3.54(1.01)	3.56(0.95)	74*(2.1)	3.55(0.98)
HDL-cholesterol mmol/l mean (SD)	1.23(0.34)	1.53(0.44)	1(0.0)	1.39(0.42)
Serum cholesterol mmol/l mean (SD)	5.51(1.13)	5.69(1.04)	1(0.0)	5.61(1.08)
Body weight kg mean (SD)	90.1(15.8)	74.1(14.5)	3(0.1)	81.8(17.0)
Diabetes^2^ n (%)	138(8.4)	106(5.8)	54(1.5)	244(7.0)
Waist circumference cm mean (SD)	101.3(11.5)	92.2(13.1)	50(1.4)	96.5(13.2)
BMI mean (SD)	28.1(4.4)	27.4(5.3)	3(0.1)	27.7(4.9)
Systolic blood pressure mmHg mean (SD)	132.2(15.9)	126.9(16.3)	2(0.1)	129.4(16.3)
Diastolic blood pressure mmHg mean (SD)	84.9(10.6)	80.7(10.0)	4(0.1)	82.7(10.5)

^1^ Self-reported

^2^Diabetes: Fasting glucose ≥ 7 mmol/l or 2-hour-glucose ≥ 12.2 mmol/L or self-reported previously diagnosed diabetes. Oral glucose tolerance testing was not performed if fasting glucose was ≥ 7mmol/l or the participant already had diagnosed diabetes. Glucose was tested on capillary plasma

*LDL-cholesterol was calculated with Friedewald’s formula, not valid if triglycerides are > 4.5 mmol/L. In individuals where triglycerides levels were > 4.5 mmol/L LDL-cholesterol was reported missing.

### Prevalence of atherosclerosis

Carotid plaques were present in 51.1% of the men and 39.0% of the women. The bilateral mean of mean cIMT was 0.68 mm in men vs. 0.64 mm in women (p < 0.001), and bilateral mean of maximal cIMT value, irrespective of side and angle, was 0.90 mm in men vs. 0.82 mm in women (p < 0.001). Overall, cIMT was higher in the left compared to the right carotid artery and was higher among men compared to women (p < 0.001).

**Table 2:** Baseline ultrasound characteristics among men and women in the VIPVIZA trial

Variables	Men (n = 1662)	Women (n = 1870)	Total (n = 3532)
Bilateral			
Plaque presence, n (%)	850(51.1)	729(39.0)	1579(44.7)
Mean of mean cIMT (SD) mm	0.68(0.13)	0.64(0.11)	0.66(0.12)
Mean of maximal cIMT (SD) mm	0.90(0.20)	0.82(0.16)	0.86(0.19)
Maximal mean value cIMT (SD) mm	0.77(0.17)	0.71(0.14)	0.74(0.16)
Left side			
Plaque presence, n (%)	635(38.2)	521(27.9)	1156(32.7)
Mean of mean cIMT (SD) mm	0.69(0.15)	0.64(0.12)	0.66(0.14)
Maximal cIMT 240° (SD) mm	0.80(0.19)	0.73(0.16)	0.76(0.17)
Mean cIMT 240° (SD) mm	0.70(0.16)	0.64(0.13)	0.67(0.15)
Maximal cIMT 210° (SD) mm	0.78(0.18)	0.73(0.15)	0.75(0.17)
Mean cIMT 210° (SD) mm	0.68(0.15)	0.64(0.13)	0.66(0.14)
Right side			
Plaque presence, n (%)	639(38.4)	509(27.3)	1148(32.5)
Mean of mean cIMT (SD) mm	0.67(0.14)	0.63(0.12)	0.65(0.13)
Maximal cIMT 120° (SD) mm	0.78(0.19)	0.73(0.15)	0.76(0.17)
Mean cIMT 120° (SD) mm	0.68(0.16)	0.64(0.13)	0.66(0.14)
Maximal cIMT 150° (SD) mm	0.76(0.17)	0.72(0.15)	0.74(0.16)
Mean cIMT 150° (SD) mm	0.66(0.14)	0.63(0.12)	0.64(0.13)

Less than 1% missing data in all variables. cIMT – Carotid Intima Media Thickness. ° Degree according to Meijer’s Arc. The cIMT measurements only include the far wall.

### Associations between CVD risk factors and ultrasound measures

The result of the multivariate analysis between risk factors and ultrasound variables is shown in Fig. 2a and b.

Among the clinical risk factors (Fig. 2a), most variables were positively associated with ultrasound variables, but physical activity, fruit and vegetable consumption, education and HDL were negatively associated with ultrasound measures. All ultrasound variables (Fig. 2b) had similar weights in the PLS analysis. However, it was observed that the bilateral variables, including cIMT values and plaque, had the highest weights, while the single cIMT measurements and plaque of the right CCA had the lowest weights.

### Contribution of CVD risk factors to the variability of ultrasound measures

Table 3 presents the risk factors that were significantly associated with different ultrasound variables in models 1–5.

**Table 3:** Associations between clinical risk factors and ultrasound variables, quantified by weights of stepwise linear regression modelling

	Model 1 (Combined_US)		Model 2 (Maximal mean cIMT bilateral)		Model 3 (Plaque)		Model 4 (Right mean cIMT)		Model 5 (Left mean cIMT)	
	*R2 = 24%*		*R2 = 18%*		*R2 = 15%*		*R2 = 14%*		*R2 = 15%*	
**Risk factor**	**Coef**	**p**	**Coef**	**p**	**Coef**	**p**	**Coef**	**p**	**Coef**	**p**
HDL-cholesterol	-0.05	**	-0.06	***	-0.07	***			-0.05	***
Diastolic blood pressure	-0.15	***	-0.13	***	-0.10	***	-0.15	***	-0.11	***
LDL-cholesterol	0.13	***	0.09	***			0.05	**	0.09	***
Systolic blood pressure	0.24	***	0.21	***	0.18	***	0.20	***	0.19	***
Antihypertensive medication	0.06	***			0.10	***				
Lipid-lowering medication	0.09	***			0.13	***				
Sex	0.18	***	0.19	***	0.12	***	0.14	***	0.17	***
S-cholesterol					0.12	***				
Education					-0.04	*				
Waist	-0.05	**			-0.10	***				
Age	0.31	***	0.29	***	0.20	***	0.28	***	0.26	***
Smoking	0.07	***	0.04	*	0.08	***			0.04	*
Geographic region	0.04	**			0.04	*				

* p < 0.05

** p < 0.01

*** p < 0.001

Only risk factors with significant contribution to the model are included here. Only complete cases, all variables present, are included in the analysis, N = 3096.

Clinical risk factors had the strongest association to the combined ultrasound measurement and explained 24% of the variability of this ultrasound variable, R2 = 24% (model 1), which was clearly stronger than the association with the variable displaying the bilateral maximal mean cIMT value, R2 = 18% (model 2). Both presence of plaque (R2 = 15%), and right mean cIMT (R2 = 14%), as well as the corresponding cIMT variable from the left side (R2 = 15%), displayed weaker associations, thus the clinical risk factors explained less of their variability (model 3–5). The pattern regarding the association of individual risk factors with ultrasound variables was similar in all five models and the risk factors with the strongest association to ultrasound variables were age, systolic blood pressure, sex, LDL and smoking. Diastolic blood pressure was negatively associated to ultrasound results. The association between CVD risk factors and the combined ultrasound measurement was similar in men and women (Table 4).

**Table 4:** Associations between clinical risk factors and the combined ultrasound measurement by gender and age using stepwise linear regression. Only risk factors with significant contribution in the models are included here

	Model 6 (Men)		Model 7 (Women)		Model 8 (40y)		Model 9 (50y)		Model 10 (60y)	
	*R2 = 20%*		*R2 = 21%*		*R2 = 22%*		*R2 = 12%*		*R2 = 12%*	
	N = 1465		N = 1631		N = 245		N = 845		N = 2006	
**Risk factor**	**Coef**	**p**	**Coef**	**p**	**Coef**	**p**	**Coef**	**P**	**Coef**	**p**
HDL-cholesterol	-0.06	**								
Diastolic blood pressure	-0.14	***	-0.16	***			-0.14	**	-0.18	***
LDL-cholesterol	0.15	***	0.12	***			0.12	***	0.16	***
Systolic blood pressure	0.21	***	0.27	***	0.10	*	0.30	***	0.25	***
Serum triglycerides					0.26	***	0.08	*		
Antihypertensive medication	0.07	**	0.05	*	0.19	**			0.08	***
Lipid-lowering medication	0.08	**	0.11	***	-0.18	**			0.12	***
Sex					0.15	*	0.22	***	0.20	***
Physically active					0.15	*				
Waist			-0.05	*			-0.12	**		
Age	0.34	***	0.29	***						
Diabetes					0.14	*				
Smoking	0.05	*	0.11	***					0.09	***
Geographic region									0.05	*

* p < 0.05

** p < 0.01

** p < 0.001

Only risk factors with significant contribution to the model are included here. Only complete cases, all variables present, are included in the analysis, N = 3096.

Risk factors explained more of the variability of the combined measurement in those aged 40 years (R = 22%), as compared to those aged 50 or 60 years (R = 12% for both). The age-specific patterns of significantly associated risk factors differed to some extent between age groups. Thus, in those aged 40 years, lipid-lowering medication was negatively associated while LDL did not associate significantly with ultrasound results. Among those aged 50 and 60 years, diastolic blood pressure was negatively and systolic blood pressure positively associated with ultrasound results.

## Discussion

The main results of our study are that during the early subclinical phase of atherosclerotic disease in a population at low/intermediate risk of CVD, clinical risk factors have a stronger association with a combined ultrasound variable (based on several measurements of cIMT on both sides and at different angles, as well as plaque present or not present), compared to single ultrasound measurements. This is in line with the recent finding of improved risk stratification in patients at high risk of CVD by the use of multi-view carotid examination including the average of several IMT measurements and the number of sites with plaques [10]. Our findings may support the integration of multi-view ultrasonography as a tool to improve CVD risk assessment also in an early phase of atherosclerosis.

### Association of CVD risk factors with ultrasound measurements

Even though CVD risk factors have a higher association with a combined variable, the risk factors only explain the variability in subclinical atherosclerosis to a limited extent. The focality of atherosclerosis could be one important reason why our results show that risk factors associate more strongly with a composite of several cIMT measurements and plaque (R = 24%) than plaque (R2 = 15%) or right mean cIMT (R2 = 14%) alone. In a previous study investigating the association of Framingham risk factors with cIMT, risk factors accounted for 28.6% of the variability in the mean CCA IMT left and right side, while age and sex contributed to 23.5% of the variability [17]. However, the study population had a higher degree of previous CVD (11%), compared to our population (2.2%), and thus more advanced atherosclerotic disease. Both the CARDIA [19] and the MESA [30] trial found a higher degree of association between risk factors and the variability of CCA IMT (R2 = 26.8% and R2 = 31%), than our combined multi-view ultrasound measurement. However, the population case mix in the CARDIA, the MESA and the VIPVIZA studies are not comparable. In the VIPVIZA study a vast majority of the participants were of white race while this was not the case in the other trials where the populations were more heterogenous. Furthermore, in the CARDIA and MESA trial a mean value of the cIMT measurements from the far and near wall of the CCA were used as compared to the far wall measurements of CCA used in VIPVIZA study.

In a study by Ku et al., traditional risk factors explained 19.5% of the variability in the plaque burden, and with the addition of less traditional risk factors, such as socioeconomic factors and white blood count, 21.9% of the variability was explained [18]. Participants in their study were older (mean age 69.4 years), had more diabetes (19%), and more anti-hypertensive and lipid-lowering treatments.

Our results are concordant with previous studies showing that the classical CVD risk factors such as, older age, male sex, low education, diabetes, high blood pressure, higher BMI, high LDL-levels, and physical inactivity result in higher risk for atherosclerosis [17, 31–34]. However, we see a different pattern of association in those aged 40 years where the association coefficient was negative with lipid lowering treatment, while there was no significant association with LDL levels. Participants aged 40 years old were relatively few and selected by having a first degree relative with CVD before the age of 60 years, that may indicate inherent factors not accounted for in the analyses. In the total population 31% were on hypertensive treatment and 11,4% were on lipid lowering treatment, among 40-year olds less than 6% were on pharmacological treatment, therefore these associations with lipid lowering and hypertensive treatment should be interpreted with caution. Furthermore, the negative association between diastolic blood pressure and ultrasound variables observed in those aged 50 and 60 years could be due to vascular stiffness and an increased pulse pressure at increasing age and atherosclerosis.

Unexpectedly, a negative association was observed between waist circumference and the combined ultrasound variable and presence of plaque. This result is not in line with previous research, where abdominal obesity is associated with subclinical atherosclerosis.

Taken together, our results correspond with the results of previous studies showing that the variation in cIMT and carotid plaque burden in a middle-aged population with subclinical atherosclerosis, to a large extent cannot be explained by traditional risk factors. This suggest that other factors may play important roles. This is also in line with results from the SCAPIS study performed partly in the same region as VIPVIZA, showing that in the general population, the positive predictive value of presence of plaque is only 45% regarding prediction of a high risk according to SCORE, i.e. ≥5% 10-year risk of CVD mortality [35].

### Prevalence of subclinical atherosclerosis

The prevalence of subclinical atherosclerosis differs between population-based studies In the ARIC study [36], 39.6% at the age of 55–59 years had plaque, compared to The REFINE-Reykjavik study with a plaque prevalence of 70.3% among men and 54.5% among women in the same age group [33]. The ACE study showed a plaque prevalence of 87% in a 63-year old population [34], and since age is the strongest risk factor for plaque, this could be part of the explanation as to why the plaque prevalence in the VIPVIZA population is in line with the ARIC population but less so with the ACE population. The definition of plaque in the REFINE study was “an isolated thickening at least two times the adjacent normal cIMT by visual assessment”, rather than the usually-applied Mannheim consensus. Different plaque definitions and population case mix make it difficult to compare studies. Furthermore, the ultrasound examination in the ARIC study was performed in 1987–1989 and ultrasound techniques have improved since then which renders it possible to detect earlier stages of atherosclerosis. The ARIC population is of a comparable age to our VIPVIZA study: 54 versus 56 years old, respectively. Blood pressure was higher in VIPVIZA compared to the ARIC population (129/82 vs. 121/73, respectively) but current smoking was lower 12.7% vs. 27%.

### Strengths and limitations of the study

A strength of the study was the overall attendance rate of 85% which is considered high in comparison to similar studies [34]. Our study population was recruited from a well described population, who display relatively high participation rates in VIP, around 65% during VIPVIZAs recruitment period, and only a small degree of social bias have been reported [37]. Therefore, our study population should fairly well represent the vast majority of the general population at low/intermediate risk, among whom the majority of CVD events occur [38], rendering our study a high relevance for public health. The large number of participants and the fact that our study was carried out within a general health care setting, rather than in a hospital or strict research setting, also renders high external validity to our study.

From a preventive perspective, it is a strength that our study population displays a relatively early and asymptomatic phase of atherosclerotic disease, suggesting that they are still at a stage where medical and lifestyle interventions can postpone or prevent future CVD events. Pictorial presentation of subclinical atherosclerosis has the potential to improve primary prevention of CVD. This was corroborated by recent publications showing a beneficial intervention effect after one and three years of follow-up [20, 39].

The potential to improve CVD risk assessment by a carotid ultrasound multi-view variable compared to single cIMT and plaque measurements will be further studied in longitudinal evaluation in the VIPVIZA trial.

The measurements of clinical risk factors were carried out in a standardized way in a stable clinical setting. A limitation is that information about use of lipid- and blood pressure-lowering treatment, and lifestyle variables, as well as education and previous myocardial infarction, were self-reported and alcohol consumption was not taken into account. In the current study carotid plaque was only determined as present or not present which limits our results. Extended analysis of plaque area and other characteristics would have given additional value to our results since plaque measurements beyond plaque prevalence are important for predictive ability [40, 41]. However, in the future 3D ultrasonography is likely to be a more reliable approach to capture the heterogeneity of subclinical carotid atherosclerosis [42].

## Conclusions

The prevalence of subclinical atherosclerosis in a low/intermediate risk population in Sweden is in line with other populations of similar age and plaque definitions. The results show that traditional risk factors for CVD are more strongly associated with a combined multi-view ultrasound variable based on multiple carotid IMT measurements and plaque presence or not than with one single measurement, suggesting that multi-view ultrasonography better captures the focality of early atherosclerotic disease. The pattern of association between traditional risk factors and subclinical atherosclerosis is similar in men and women. Even though CVD risk factors have a higher association with a combined variable, risk factors only explain the variability in subclinical atherosclerosis to a limited extent.

## Electronic supplementary material

Below is the link to the electronic supplementary material.

Supplementary Material 1

## References

[1] Mortality GBD, Causes of Death C (2016). Global, regional, and national life expectancy, all-cause mortality, and cause-specific mortality for 249 causes of death, 1980–2015: a systematic analysis for the global burden of Disease Study 2015. Lancet.

[2] O’Donnell MJ, Xavier D, Liu L, Zhang H, Chin SL, Rao-Melacini P, Rangarajan S, Islam S, Pais P, McQueen MJ, Mondo C, Damasceno A, Lopez-Jaramillo P, Hankey GJ, Dans AL, Yusoff K, Truelsen T, Diener HC, Sacco RL, Ryglewicz D, Czlonkowska A, Weimar C, Wang X, Yusuf S (2010) investigators I Risk factors for ischaemic and intracerebral haemorrhagic stroke in 22 countries (the INTERSTROKE study): a case-control study. Lancet 376:112 – 23. 10.1016/S0140-6736(10)60834-3 10.1016/S0140-6736(10)60834-3 20561675

[3] Yusuf S, Hawken S, Ounpuu S, Dans T, Avezum A, Lanas F, McQueen M, Budaj A, Pais P, Varigos J, Lisheng L, Investigators IS (2004). Effect of potentially modifiable risk factors associated with myocardial infarction in 52 countries (the INTERHEART study): case-control study. Lancet.

[4] Bauer M, Delaney JA, Mohlenkamp S, Jockel KH, Kronmal RA, Lehmann N, Mukamal KJ, Moebus S, Polak JF, Dragano N, Budoff MJ, Erbel R, McClelland RL, Investigator Group of the Heinz Nixdorf Recall S (2013) Multi-Ethnic Study of A, Comparison of factors associated with carotid intima-media thickness in the Multi-ethnic Study of Atherosclerosis (MESA) and the Heinz Nixdorf Recall Study (HNR). Journal of the American Society of Echocardiography 26:667 – 73. 10.1016/j.echo.2013.03.011 10.1016/j.echo.2013.03.011 PMC3694173 23611058

[5] Bian L, Xia L, Wang Y, Jiang J, Zhang Y, Li D, Li W, He Y (2018). Risk factors of subclinical atherosclerosis and plaque burden in high risk individuals: results from a community-based study. Front Physiol.

[6] Nilsson Wadstrom B, Engstrom G, Nilsson PM (2021). Exploring and comparing definitions of healthy vascular ageing in the population: characteristics and prospective cardiovascular risk. J Hum Hypertens.

[7] Fernandez-Alvarez V, Linares Sanchez M, Lopez Alvarez F, Suarez Nieto C, Makitie AA, Olsen KD, Ferlito A (2022). Evaluation of Intima-Media thickness and arterial stiffness as early ultrasound biomarkers of carotid artery atherosclerosis. Cardiol Ther.

[8] Gepner AD, Young R, Delaney JA, Tattersall MC, Blaha MJ, Post WS, Gottesman RF, Kronmal R, Budoff MJ, Burke GL, Folsom AR, Liu K, Kaufman J, Stein JH (2015) Comparison of coronary artery calcium presence, carotid plaque presence, and carotid intima-media thickness for cardiovascular disease prediction in the multi-ethnic study of atherosclerosis. Circulation Cardiovasc imaging 8. 10.1161/CIRCIMAGING.114.002262 10.1161/CIRCIMAGING.114.002262 PMC4299916 25596139

[9] Paraskevas KI, Sillesen HH, Rundek T, Mathiesen EB, Spence JD (2020). Carotid intima-media thickness Versus Carotid Plaque Burden for Predicting Cardiovascular Risk. Angiology.

[10] Georgiopoulos G, Mavraganis G, Delialis D, Georgiou S, Aivalioti E, Patras R, Petropoulos I, Dimopoulou MA, Angelidakis L, Sianis A, Bampatsias D, Dimoula A, Maneta E, Kosmopoulos M, Vardavas C, Stellos K, Stamatelopoulos K (2022). Carotid ultrasonography improves residual risk stratification in guidelines-defined high cardiovascular risk patients. Eur J Prev Cardiol.

[11] Naqvi TZ, Lee MS (2014). Carotid intima-media thickness and plaque in cardiovascular risk assessment. JACC Cardiovasc imaging.

[12] Polak JF, O’Leary DH (2016). Carotid intima-media thickness as surrogate for and predictor of CVD. Glob Heart.

[13] Stein JH, Korcarz CE, Hurst RT, Lonn E, Kendall CB, Mohler ER, Najjar SS, Rembold CM, Post WS, Task F, American Society of Echocardiography Carotid Intima-Media Thickness (2008). Use of carotid ultrasound to identify subclinical vascular disease and evaluate cardiovascular disease risk: a consensus statement from the American Society of Echocardiography Carotid Intima-Media Thickness Task Force. Endorsed by the Society for Vascular Medicine. J Am Soc Echocardiogr.

[14] Luo X, Yang Y, Cao T, Li Z (2011). Differences in left and right carotid intima-media thickness and the associated risk factors. Clin Radiol.

[15] Ma SM, Wei CK, Liang CC, Chou JM, Lee SY (2011). The age correlation of the carotid intima-media thickness according to sex and side in asymptomatic subjects. Acta Neurol Taiwan.

[16] Peters SA, den Ruijter HM, Palmer MK, Grobbee DE, Crouse JR 3rd, O’Leary DH, Evans GW, Raichlen JS, Bots ML, Investigators MS (2012) Extensive or restricted ultrasound protocols to measure carotid intima-media thickness: analysis of completeness rates and impact on observed rates of change over time. J Am Soc Echocardiogr 25:91–100. 10.1016/j.echo.2011.09.009 10.1016/j.echo.2011.09.009 21982737

[17] Polak JF, Pencina MJ, Meisner A, Pencina KM, Brown LS, Wolf PA, D’Agostino RBS (2010). Associations of carotid artery intima-media thickness (IMT) with risk factors and prevalent cardiovascular disease: comparison of mean common carotid artery IMT with maximum internal carotid artery IMT. J Ultrasound Med.

[18] Kuo F, Gardener H, Dong C, Cabral D, Della-Morte D, Blanton SH, Elkind MS, Sacco RL, Rundek T (2012). Traditional cardiovascular risk factors explain the minority of the variability in carotid plaque. Stroke.

[19] Polak JF, Person SD, Wei GS, Godreau A, Jacobs DR Jr, Harrington A, Sidney S, O’Leary DH (2010) Segment-specific associations of carotid intima-media thickness with cardiovascular risk factors: the coronary artery Risk Development in Young adults (CARDIA) study. Stroke 41:9–15. 10.1161/STROKEAHA.109.566596 10.1161/STROKEAHA.109.566596 PMC3163306 19910544

[20] Naslund U, Ng N, Lundgren A, Fharm E, Gronlund C, Johansson H, Lindahl B, Lindahl B, Lindvall K, Nilsson SK, Nordin M, Nordin S, Nyman E, Rocklov J, Vanoli D, Weinehall L, Wennberg P, Wester P, Norberg M, group Vt (2019). Visualization of asymptomatic atherosclerotic disease for optimum cardiovascular prevention (VIPVIZA): a pragmatic, open-label, randomised controlled trial. Lancet.

[21] Norberg M, Wall S, Boman K, Weinehall L (2010) The Vasterbotten Intervention Programme: background, design and implications. Global health action 3. 10.3402/gha.v3i0.4643 10.3402/gha.v3i0.4643 PMC2844807 20339479

[22] Alberti KG, Zimmet PZ (1998). Definition, diagnosis and classification of diabetes mellitus and its complications. Part 1: diagnosis and classification of diabetes mellitus provisional report of a WHO consultation. Diabet Med.

[23] Friedewald WT, Levy RI, Fredrickson DS (1972) Estimation of the concentration of low-density lipoprotein cholesterol in plasma, without use of the preparative ultracentrifuge. Clin Chem. 1972 Jun;18(6):499–502 4337382

[24] Touboul PJ, Hennerici MG, Meairs S, Adams H, Amarenco P, Bornstein N, Csiba L, Desvarieux M, Ebrahim S, Fatar M, Hernandez Hernandez R, Jaff M, Kownator S, Prati P, Rundek T, Sitzer M, Schminke U, Tardif JC, Taylor A, Vicaut E, Woo KS, Zannad F, Zureik M (2007) Mannheim carotid intima-media thickness consensus (2004–2006). An update on behalf of the Advisory Board of the 3rd and 4th Watching the Risk Symposium, 13th and 15th European Stroke Conferences, Mannheim, Germany, 2004, and Brussels, Belgium, 2006. Cerebrovasc Dis 23:75–80. 10.1159/000097034 10.1159/000097034 17108679

[25] Nyman E, Vanoli D, Naslund U, Gronlund C (2020). Inter-sonographer reproducibility of carotid ultrasound plaque detection using Mannheim consensus in subclinical atherosclerosis. Clin Physiol Funct Imaging.

[26] Vanoli D, Lindqvist P, Wiklund U, Henein M, Naslund U Fully automated on-screen carotid intima-media thickness measurement: a screening tool for subclinical atherosclerosis.Journal of clinical ultrasound41:333–9. 10.1002/jcu.22041 10.1002/jcu.22041 23553729

[27] Krishnan A, Williams LJ, McIntosh AR, Abdi H (2011). Partial least squares (PLS) methods for neuroimaging: a tutorial and review. NeuroImage.

[28] Thorsson B, Eiriksdottir G, Sigurdsson S, Gudmundsson EF, Bots ML, Aspelund T, Arntzen KA, Mathiesen EB, Gudnason V (2018). Population distribution of traditional and the emerging cardiovascular risk factors carotid plaque and IMT: the REFINE-Reykjavik study with comparison with the Tromso study. BMJ open.

[29] Zeb I, Budoff MJ (2011). MESA: the NIH-sponsored study that validates atherosclerosis imaging for primary prevention. Curr Atheroscler Rep.

[30] Polak JF, Szklo M, O’Leary DH Associations of Coronary Heart Disease with Common Carotid Artery Near and Far Wall Intima-Media thickness: the multi-ethnic study of atherosclerosis.Journal of the American Society of Echocardiography28:1114–21. 10.1016/j.echo.2015.04.001 10.1016/j.echo.2015.04.001 PMC4567434 25944425

[31] Barnett PA, Spence JD, Manuck SB, Jennings JR (1997). Psychological stress and the progression of carotid artery disease. J Hypertens.

[32] Zanchetti A, Bond MG, Hennig M, Neiss A, Mancia G, Dal Palu C, Hansson L, Magnani B, Rahn KH, Reid J, Rodicio J, Safar M, Eckes L, Ravinetto R (1998). Risk factors associated with alterations in carotid intima-media thickness in hypertension: baseline data from the european Lacidipine study on atherosclerosis. J Hypertens.

[33] Sturlaugsdottir R, Aspelund T, Bjornsdottir G, Sigurdsson S, Thorsson B, Eiriksdottir G, Gudnason V (2016). Prevalence and determinants of carotid plaque in the cross-sectional REFINE-Reykjavik study. BMJ open.

[34] Ihle-Hansen H, Vigen T, Ihle-Hansen H, Ronning OM, Berge T, Thommessen B, Lyngbakken MN, Orstad EB, Enger S, Nygard S, Rosjo H, Tveit A (2018) Prevalence of Carotid Plaque in a 63- to 65-Year-old norwegian cohort from the General Population: the ACE (Akershus Cardiac examination) 1950 study. J Am Heart Association 7. 10.1161/JAHA.118.008562 10.1161/JAHA.118.008562 PMC6015330 29739796

[35] Ostgren CJ, Soderberg S, Festin K, Angeras O, Bergstrom G, Blomberg A, Brandberg J, Cederlund K, Eliasson M, Engstrom G, Erlinge D, Fagman E, Hagstrom E, Lind L, Mannila M, Nilsson U, Oldgren J, Ostenfeld E, Persson A, Persson J, Persson M, Rosengren A, Sundstrom J, Swahn E, Engvall JE, Jernberg T (2020). Systematic coronary risk evaluation estimated risk and prevalent subclinical atherosclerosis in coronary and carotid arteries: a population-based cohort analysis from the swedish cardiopulmonary Bioimage Study. Eur J Prev Cardiol.

[36] Li R, Duncan BB, Metcalf PA, Crouse JR 3rd, Sharrett AR, Tyroler HA, Barnes R, Heiss G (1994) B-mode-detected carotid artery plaque in a general population. Atherosclerosis risk in Communities (ARIC) Study investigators. Stroke 25:2377–2383. 10.1161/01.str.25.12.2377 10.1161/01.str.25.12.2377 7974576

[37] Norberg M, Blomstedt Y, Lonnberg G, Nystrom L, Stenlund H, Wall S, Weinehall L (2012). Community participation and sustainability–evidence over 25 years in the Vasterbotten intervention Programme. Global health action.

[38] Polonsky TS, Greenland P (2012). CVD screening in low-risk, asymptomatic adults: clinical trials needed. Nat reviews Cardiol.

[39] Bengtsson A, Norberg M, Ng N, Carlberg B, Gronlund C, Hultdin J, Lindahl B, Lindahl B, Nordin S, Nyman E, Wennberg P, Wester P, Naslund U (2021). The beneficial effect over 3 years by pictorial information to patients and their physician about subclinical atherosclerosis and cardiovascular risk: results from the VIPVIZA randomized clinical trial. Am J Prev Cardiol.

[40] Spence JD, Eliasziw M, DiCicco M, Hackam DG, Galil R, Lohmann T (2002). Carotid plaque area: a tool for targeting and evaluating vascular preventive therapy. Stroke.

[41] Herder M, Johnsen SH, Arntzen KA, Mathiesen EB (2012). Risk factors for progression of carotid intima-media thickness and total plaque area: a 13-year follow-up study: the Tromso Study. Stroke.

[42] Fenster A, Landry A, Downey DB, Hegele RA, Spence JD (2004). 3D ultrasound imaging of the carotid arteries. Curr Drug Targets Cardiovasc Haematol Disord.

