# Supplementary material for: Multi-view carotid ultrasound is stronger associated with cardiovascular risk factors than presence of plaque or single carotid intima media thickness measurements in subclinical atherosclerosis
Source: Int J Cardiovasc Imaging. 2023 May 30;39(8):1461–71. doi: 10.1007/s10554-023-02868-0 (PMC10427531; doi:10.1007/s10554-023-02868-0)
Supplement: Supplementary file 1 — Supplementary Material 1 [file 10554_2023_2868_MOESM1_ESM.docx]

Supplementary material

1.


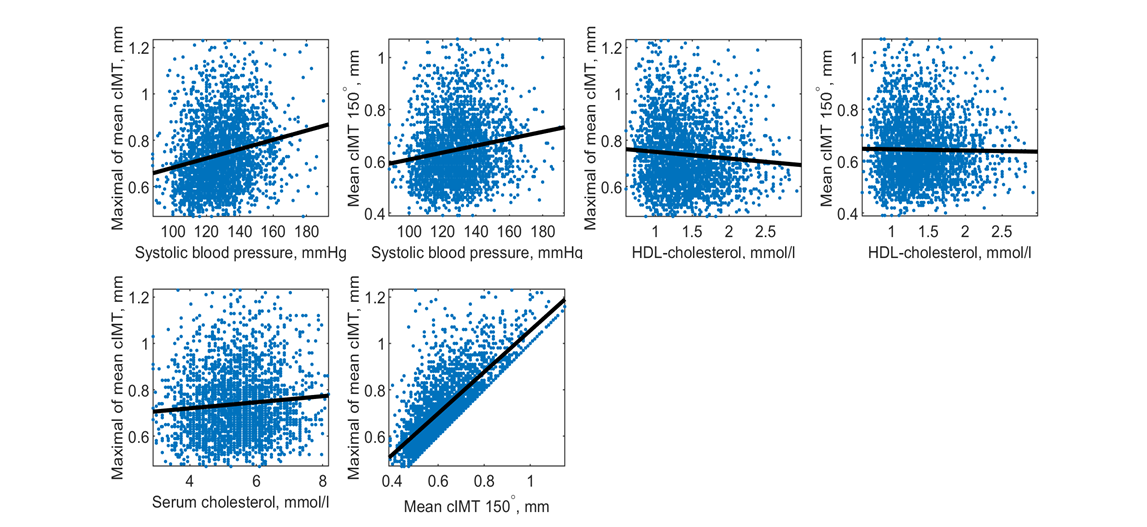


Supplementary material 1. Examples of scatterplots between risk factors, systolic blood pressure, HDL-cholesterol and serum cholesterol and ultrasound variables Maximal of mean cIMT and Mean cIMT 150^o^, and between the two ultrasound variables
